# Simultaneous Development of Acute Acquired Concomitant Esotropia in Two Siblings during the COVID-19 Pandemic: A Case Report

**DOI:** 10.22599/bioj.283

**Published:** 2023-02-06

**Authors:** Danielle Carter, Priyanka Pujara, Kate Bolton, Rory Nicholson

**Affiliations:** 1 Portsmouth Hospitals University NHS Trust, GB

**Keywords:** Esotropia, COVID-19, Lockdown, strabismus, acute acquired concomitant esotropia

## Abstract

**Aim:**

To report a case of two siblings who near–simultaneously developed a large angle concomitant esotropia during the COVID–19 pandemic, and to describe their treatment and outcomes.

**Method:**

A 5–year–old boy and his 11–year–old sister were presented to the hospital eye service in early 2021, having both developed acute–onset large angle esotropia within three months of each other. Neither had any significant past medical, ophthalmic, or family history. The siblings lived in the same household, and both experienced lifestyle changes as a result of the UK lockdown in response to COVID–19.

**Results:**

Each sibling was treated with right medial rectus recession (5.5 mm) and right lateral rectus resection (7 mm), and at a three–month follow–up, both were minimally esophoric with restored binocularity.

**Conclusion:**

The unusual and abrupt changes in lifestyle imposed by the COVID–19 pandemic highlight the likelihood of an environmental aetiology for some forms of esotropia and raise the possibility that extended screen time may be a contributory factor.

## Introduction

In March 2020, the COVID–19 (coronavirus disease 2019) pandemic led to the introduction of national lockdowns across the United Kingdom. On March 16, the British government advised the public to work at home where possible and to avoid social gatherings. On March 20, UK schools, pubs, restaurants, and social venues were closed with recommendations to stay at home, except for essential movement. ‘Stay at home’ lockdowns began on March 23. Work and education abruptly shifted to a remote, online model, with more time spent indoors and in front of a screen. These measures remained in effect in England until March 8, 2021, when schools re–opened for face–to–face education.

Amongst the authors and many of their colleagues across the nation, the perception is that there has been an increased frequency of children and young adults presenting with acute onset non–accommodative esotropia following the national lockdown, with speculation that this may be related to increased time spent indoors looking at a screen. We are, however, not aware of any studies demonstrating such a phenomenon to date in the United Kingdom.

This article reports the case of two siblings in the same household who simultaneously developed large angle concomitant esotropia during the COVID–19 UK national lockdown.

## Case Reports

### Sibling 1

A 5-year-old boy developed a right esotropia over a period of several weeks in January 2021. He was in good general health, with no significant past medical history. He was systemically well and did not report double vision, headaches, nausea, or vomiting. There was no known family history of strabismus; the only reported family history was that both parents were myopic. He had been spending significantly more time in front of a screen over the pandemic, due to online education and national restrictions on meeting others outside the family bubble.

He was seen by a local optometrist in March 2021 and found to be mildly hypermetropic on cycloplegic retinoscopy (OD +1.50, OS +1.25). Glasses were prescribed and he was referred to the hospital eye service.

He was seen by an orthoptist in May 2021. Unaided visual acuity was OD 0.08, OS 0.06 LogMAR. With glasses, visual acuity was 0.2 LogMAR in each eye, and it was noted that he tended to look over the top of the glasses. He had a moderate right esotropia, initially maintained straight by corneal light reflex, and glasses had no effect on his control or the angle of deviation. With and without the glasses, he measured 45/50 prism dioptres (Δ) base out at near and 45Δ base out at distance. Binocular function was not proven during this visit, although the patient’s history suggested previous binocular function. He had full ocular motility, and no cranial nerve defect. Examination of anterior and posterior segments was unremarkable.

The boy’s mother mentioned that since his referral from the optician, his 11-year-old sister had also developed a convergent squint and was due to be seen at the hospital in two weeks’ time.

### Sibling 2

An 11-year-old girl was seen for an emergency appointment at her local opticians in April 2021 due to sudden onset diplopia. She was in good general health, with no significant ophthalmic or medical history. Like her brother, she had been spending significant time in front of a screen during the national lockdown.

The optician found no refractive error and documented a ‘small concomitant right esotropia’. A referral was made to the hospital eye service, and she was seen by an orthoptist approximately six weeks later.

At her orthoptic appointment, unaided visual acuity measured 0.00 LogMAR in each eye. A cover test revealed an esophoria which broke down into a right esotropia immediately on dissociation. The esotropia measured 30Δ base out at near and 20Δ base out at distance. She demonstrated 170” of arc stereoacuity using the Frisby Stereotest. Ocular motility was full, and further assessment of cranial nerves, anterior, and posterior segments was unremarkable. Cycloplegic retinoscopy confirmed no significant refractive error (OD plano/–0.25 × 180, OS plano/–0.25 × 090).

## Results

Both siblings were seen for a follow-up appointment in September 2021. Both now demonstrated a constant large angle esotropia—sibling 1: 54Δ base out at near and 45Δ base out at distance (without glasses), sibling 2: 45Δ base out at near and 40/45Δ base out at distance.

The siblings were listed for strabismus surgery, and this was carried out in November 2021 on the same operating list. The rectus muscle insertions were found at their usual anatomic locations. Both had a right medial rectus recession (5.5 mm) and right lateral rectus resection (7 mm) on fixed 6/0 polyglactin 910 sutures.

**Figure 1:**
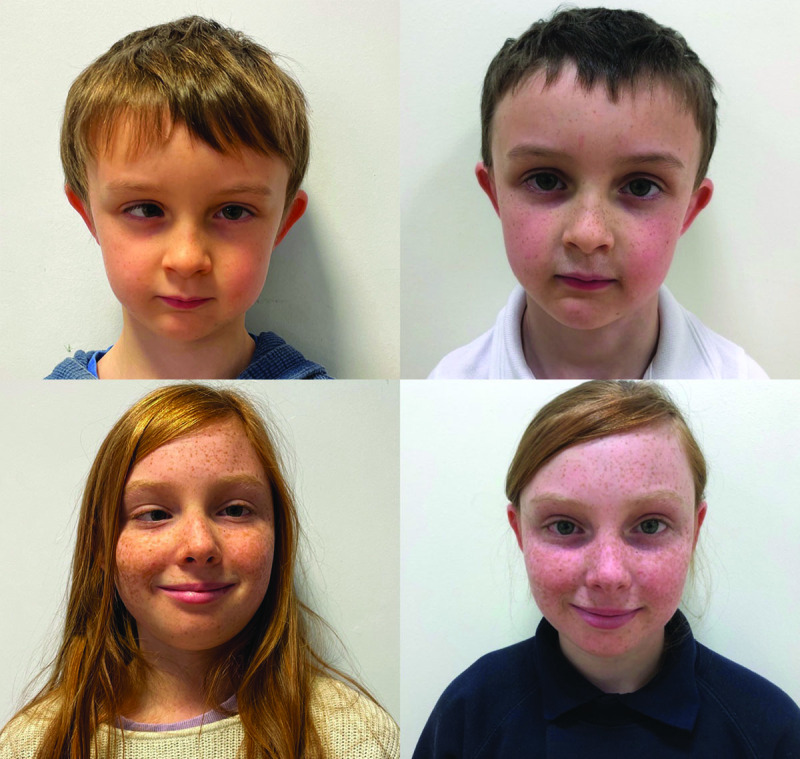
Sibling 1 (top row) and sibling 2 (bottom row) pre–operatively and at three–months post–operatively. All images in primary position.

At the three-month post-operation mark, both siblings were esophoric with restored binocularity: sibling 1 achieving 100” of arc stereoacuity and sibling 2 achieving 40” of arc stereoacuity using the Randot Stereotest. Table 1 presents their PCT measurements at each visit, and Figure 1 shows cosmesis pre-operative and at the 3-month post-operative visits.

**Table 1:** Angle of deviation at presentation, pre–operatively, two weeks post–operatively, and three–months post–operatively. ET = esotropia, E(T) = intermittent esotropia, EP = esophoria.


VISIT	SIBLING 1		SIBLING 2	
	
	NEAR	DISTANCE	NEAR	DISTANCE

Presentation	45/50Δ ET	45Δ ET	30Δ E(T)	20Δ E(T)

Pre–operative	54Δ ET	45Δ ET	45Δ ET	45Δ ET

2 weeks post–operative	16Δ E(T)	4Δ EP	14Δ EP	8Δ EP

3 months post–operative	10Δ EP	4Δ EP	6Δ EP	1Δ EP


## Discussion

The aetiology of basic (non-accommodative) esotropia is not well understood, but it is felt to have both genetic and environmental components. Twin studies have previously suggested that environmental factors are sufficient in causing most phorias, whereas genetic susceptibility is necessary to develop manifest strabismus (Wilmer & Backus 2009).

Acute acquired concomitant esotropia (AACE) is a recognised entity characterised by the rapid onset of large angle deviation in all directions of gaze—an issue that may be present intermittently before becoming constant—in the absence of underlying neurological disease (Burian & Miller 1958; Hoyt & Good 1995). It is associated with good binocular potential. The two cases described in this report are consistent with Type 2 (Franceschetti type) AACE, as neither sibling has significant refractive error or accommodative elements.

Cases of AACE manifesting in siblings have previously been reported, although such reports are rare. Norbis and Malbran (1956) reported a case series of four siblings who all developed a late–onset concomitant esotropia with diplopia, although not simultaneously. Buch and Vinding have reported the largest case series of AACE among children and described it as a ‘relatively rare condition’ occurring in children of all ages, identifying 48 cases over a 13–year period (Buch & Vinding 2015).

Anecdotally, the perception amongst our colleagues nationally is that there has been an increased frequency of children and young adults presenting with AACE following the COVID–19 lockdown, but this has not been demonstrated in any large–scale studies to date.

Internationally there have been case reports published of AACE occurring in relation to increased screen usage during the pandemic. An article published during the first year of the pandemic documents a case series of AACE in four Italian children, who had been engaging in over eight hours of screen time each day (Vagge *et al.* 2020). Mohan *et al*. (2021), collected over a two–month period, reported 8 cases of AACE in Indian children attending online classes for over 4 hours a day. This study also highlighted how the incident differed from the two–month period prior to lockdown (January–February 2020), in which only one case was reported. Neither article reports on the management of these children nor the outcomes of their treatment. Neena *et al*. (2022) conducted a retrospective study of 15 patients in South Asia who presented with AACE during the COVID–19 pandemic, for which the mean duration of near activity per day was 8.6 hours.

Prior to the pandemic, cases of AACE linked to excessive smartphone use had previously been described. Lee *et al*. (2016) reported the development of AACE in 12 teenagers who engaged in smartphone use for over four hours a day. The majority of these cases were myopic. Three of these patients ultimately underwent strabismus surgery and regained binocular single vision.

Abnormalities of accommodative convergence amplitudes have been proposed as one mechanism underlying the development of AACE. Lee *et al*. (2016) proposed that dynamic preponderance of the medial rectus muscles after sustained near point fixation may be the underlying mechanism for the development of AACE in their largely myopic case series, consistent with Bielschowsky’s theory. However, the Burian–Franceschetti type is not thought to be related to excessive accommodative convergence, and has instead been linked to reduced divergent fusional amplitudes (Campos 2008). It is possible that both mechanisms may coexist simultaneously in emmetropic patients who have been engaging in excessive near–point fixation — indeed the authors feel that this is likely to be the case.

There has been significant debate about the potential risks of excessive screen time, and to what extent this should be limited for children. Screen time has been postulated as a cause for increasing rates of myopia in children, although this was not supported by a recent systematic review (Lanca & Saw 2020). A review of the evidence (Stiglic & Viner 2019) led the Royal College of Paediatrics and Child Health to conclude that ‘the risks from screen exposure should not be overstated. The evidence is relatively weak overall. Further, the magnitude of impact of screens is small on key health outcomes’. Furthermore, it was argued during the pandemic that access to opportunities for ‘digital play’, online socialising with friends and family, and continued education was essential for supporting the wellbeing and development of young people (Livingstone & Pothong 2022).

However, there is evidence to suggest that modifying screen use behaviour may be beneficial in cases of AACE. In the study described above, Lee *et al*. (2016) found that withholding smartphone use for one month reduced the size of esodeviation in all of the 12 cases of AACE they observed—seven of whom did not require any surgical intervention and achieved binocular single vision at follow–up. Reduction of near work contributed to the improvement of five patients when used alongside divergence exercises in the study by Neena *et al*. (2022). Viewing distance may also be relevant; Lee *et al*. commented that all their cases of AACE viewed smartphones at a close distance of 30 cm or less, and Mohan *et al*. also reported that all their cases were viewing their smartphones at a distance of 30 cm or less. The digital device habits of 10 children with AACE were compared with age–matched controls in another study (Van Hoolst *et al.* 2022). There was a statistically significant reduction in working distance of the 10 cases with AACE in comparison to the controls. There was no statistically significant difference between the two groups for screen time of more than two hours per day and use of devices in the bedroom.

Whilst favourable outcomes of AACE treated with strabismus surgery is well recognised (Lee *et al.* 2016; Hayashi *et al.* 2022; Neena *et al.* 2022; Wu *et al.* 2022), conservative approaches have also been trialled. Wu *et al*. (2022) evaluated the role of step–by–step increase of Fresnel Prism power for the treatment of smartphone associated AACE causing esodeviations of 25Δ and less. It was successful in inducing binocular single vision within a year for 14 of 36 patients, and it also showed some reduction in the angle of deviation for 20 patients who continued prism wearing beyond one year. Two patients underwent strabismus surgery for failed prismatic treatment. Wu *et al*. (2022) found that treatment is more likely to be successful in patients with a shorter duration between onset and treatment, and in patients with smaller angles of deviation.

Hayashi *et al*. (2022) recognised that cases of sustained AACE are difficult to treat. They assessed the role of topical cycloplegics in smartphone associated AACE that has been untreated for more than one year by conducting a case–control study. 13 of 14 patients in the treatment group had resolution of diplopia using cycloplegics, either alone (four patients) or alongside treatment with built–in prism spectacles (nine patients). Similarly to Wu *et al*. (2022), Hayashi *et al*. (2022) found that there was a negative correlation between a longer duration from onset to treatment and the effectiveness of treatment.

There are limitations in this case report which we have considered when drawing conclusions. The clinical assessment of the two siblings was restricted by the COVID–19 pandemic, which limited hospital appointment times and patient–clinician contact. It is not known exactly how much time was spent indoors, or in front of a screen by either sibling prior to presentation. Since they were in full–time education, which was conducted virtually, digital device usage was likely to have been considerable. Phoric status prior to onset of AACE is not known for either sibling, and prism fusion range and accommodative convergence/accommodation ratio was not recorded before or after treatment. Prior binocularity was assumed due to the presentation with symptomatic diplopia, evidence of sensory fusion and stereopsis, and a demonstration of good postoperative binocular function. Due to the large manifest angle and minimal refractive error, neither prisms nor cycloplegia were trialled in either sibling.

We suggest that although it may not be practical to withhold all screen use in children, it may be advisable to limit the total hours of screen time per day, and to avoid viewing screens from a distance closer than 30 cm. Conservative treatment with cycloplegics (with or without prisms) may form part of the initial therapy within the first three months. Fresnel Prisms may also be suitable in patients presenting within one year of onset with small angles of deviations of 25Δ or less. In patients with persistent diplopia or large angle deviations, strabismus surgery is recommended.

## Conclusion

This case report provides support for an environmental aetiology for acute acquired concomitant esotropia in childhood, in addition to a possible inherited component. Increased screen use— particularly at short viewing distances—should be considered a significant factor in the aetiology of acute acquired concomitant esotropia.

Standard surgical management has so far achieved excellent visual outcomes in the two cases described.
